# Evaluation of Relationship between Serum Liver Enzymes and Hypertension: A Cross-Sectional Study Based on Data from Rafsanjan Cohort Study

**DOI:** 10.1155/2022/5062622

**Published:** 2022-04-14

**Authors:** Parvin Khalili, Saeedeh Abdollahpoor, Fatemeh Ayoobi, Alireza Vakilian, Hamid Hakimi, Zohreh Rajabi, Zahra Jamali

**Affiliations:** ^1^Social Determinants of Health Research Centre, Rafsanjan University of Medical Sciences, Rafsanjan, Iran; ^2^Department of Epidemiology, School of Public Health, Rafsanjan University of Medical Sciences, Rafsanjan, Iran; ^3^Growth and Development Research Center, Tehran University of Medical Sciences, Tehran, Iran; ^4^Occupational Safety and Health Research Center, NICICO, World Safety Organization and Rafsanjan University of Medical Sciences, Rafsanjan, Iran; ^5^Non-Communicable Diseases Research Center, Rafsanjan University of Medical Sciences, Rafsanjan, Iran; ^6^Neurology Department, School of Medicine, Rafsanjan University of Medical Sciences, Rafsanjan, Iran; ^7^Immunology of Infectious Diseases Research Center, Rafsanjan University of Medical Sciences, Rafsanjan, Iran

## Abstract

**Background:**

Hypertension as a major risk factor for cardiovascular diseases is among the leading causes of death worldwide. The relationship between elevated serum levels of liver enzymes and hypertension has been reported in limited studies, and to the best of our knowledge, there are no previous reports in the literature on this issue in the southeast of Iran. Our investigation aimed at evaluating the relation between ALT, AST, GGT, and ALP with hypertension in the Rafsanjan Cohort Study, a city in Kerman Province, Iran.

**Methods:**

In this cross-sectional study, we used data obtained from the Rafsanjan Cohort Study (RCS), as a part of the prospective epidemiological research studies in Iran (PERSIAN). The association of the liver enzymes levels with hypertension was investigated using the multivariable logistic regression models.

**Results:**

Among 9930 participants, the mean age (±SD) was 49.94 (±9.56) years, and 46.56% were men. The odds of abnormal blood pressure significantly increased along with the higher levels of ALT, GGT, and ALP which remained significant only for ALP after adjustment for all confounding variables in both males and females (OR in males: 1.36, 95% CI = 1.09–1.69, OR in females: 1.25, 95% CI = 1.01–1.54). In subjects with normal levels of ALT, AST, GGT, and ALP, dose-response increases were observed for abnormal blood pressure in both genders. Finally, we found that, among liver enzymes, only elevated ALP was significantly correlated with the odds of stage 1 hypertension and stage 2 hypertension for both genders.

**Conclusions:**

In subjects with normal levels of ALT, AST, GGT, and ALP, dose-response increases were observed for abnormal blood pressure in both genders. Increased serum ALP activity was positively associated with increased odds of hypertension in males and females. Therefore, increased ALP could be an early indicator of hypertension.

## 1. Introduction

Hypertension is one of the major causes of premature death globally. Estimates suggest that the prevalence of hypertension in low- and middle-income countries (31.5%) was higher than that in high-income countries (28.5%) [1]. Hypertension increases with high sodium and low potassium intake, obesity, alcohol consumption, physical inactivity, and unhealthy diet as modifiable risk factors. Factors such as the family history of hypertension, age over 65 years, and the presence of diabetes and chronic renal diseases are grouped as nonmodifiable risk factors. The prevalence of hypertension is variable due to variations in the levels of these risk factors. Early detection and management of hypertension in order to reduce premature mortality in high-risk populations is an emergency need for human societies [2].

There is an increasing correlation between liver dysfunction and high blood pressure. The liver plays an important role in many metabolic functions such as protein production, blood clotting, cholesterol biosynthesis, glucose, and iron metabolism. The liver enzymes such as alanine aminotransferase (ALT), aspartate aminotransferase (AST), *γ*-glutamyl transferase (GGT), and alkaline phosphatase (ALP) are routinely screened for evaluation of liver function [3]. The levels of ALT and AST are increased in the plasma due to numerous medical conditions including nonalcoholic fatty liver disease (NAFLD) [4] as the most common cause of elevated liver enzymes [5]. GGT is a marker of alcohol consumption, but it is also related to the fat precipitation in the liver (fatty liver) [6]. Some studies have reported an association between higher serum GGT levels and hypertension [3, 7, 8] which was in contrast to the study done by Forlani et al. [9]. Previous studies reported that there is an association between higher ALP and ALT levels and hypertension [10–12]. However, there are studies that do not support this association [3, 8, 9].

As mentioned above, previous studies reported a wide variation in the prevalence of increased liver enzymes in hypertension that may be due to different reference values, age range, sex, race, and demographics [2, 3, 8, 10, 11]. The epidemiological data concerning the extent of elevated liver enzymes in Iranian hypertensive individuals are limited. Since early detection of hypertension is of great importance, finding a clear association between this disorder and liver enzymes may be helpful for the earlier prognosis of hypertension. Due to the enhanced prevalence of hypertension globally [13] and also the lack of previous studies in this field in the southeast of Iran, the purpose of this study was to investigate the relationship between liver enzyme levels and hypertension in a large sample size in the adult population of the southeast of Iran.

## 2. Methods

### 2.1. Study Design and Patient Selection

This cross-sectional study was conducted using the Rafsanjan Cohort Study (RCS) data, as a part of the prospective epidemiological research studies in Iran (PERSIAN) [14]. Briefly, RCS is a cohort population-based study that was initiated in August 2015 in Rafsanjan, a region in the southeast of Iran. A total number of 9991 individuals aged 35–70 years from both genders willingly participated and signed the informed written consent letter [15].

The protocol of the study was designed according to the PERSIAN, and it was approved by the Ethics Committee of Rafsanjan University of Medical Sciences (Ethical codes: ID: IR.RUMS.REC.1400.002).

### 2.2. Data Collection

All participants underwent a standardized interview to complete validated questionnaires containing demographic data, socioeconomic status, smoking behavior, opium use, alcohol consumption, medical history, nutrition, and physical activity. Anthropometric measurements were done for all participants. Blood pressure was taken two times in each arm, and the average of the second measurement in the right and left arms was used to report blood pressure. The first measurement was taken in the sitting position, after at least 5 minutes of rest. The second measurement was taken in the sitting position, at least 10 minutes after the first measurement. Blood pressure readings are expressed in millimeters of mercury (mmHg). Socioeconomic status was also determined using the wealth score index (WSI). The WSI was calculated by multiple correspondence analysis (MCA) of the subjects' economic and social variables [16]. According to WSI, the studied population was divided into four groups: low class, low-middle class, middle-high class, and high class. To evaluate the intensity of physical activity, metabolic equivalent of task (MET) was used. Physical activity was assessed according to the 24 h physical activity and a 22- item questionnaire and was categorized as low (≤35.29 MET-hours per week), moderate (35.30–40.32 MET-hours per week), and heavy (≥40.32 MET-hours per week), respectively, based on the 25th and 75th percentile [16]. Questionnaires were validated by the PERSIAN [14].

Blood samples were taken between 7:00 AM and 9:00 AM, after 12–14 h of fasting. Fasting blood sugar (FBS), total cholesterol, high-density lipoprotein cholesterol (HDL cholesterol), low-density lipoprotein cholesterol (LDL cholesterol), triglycerides (TG), SGOT (AST), SGPT (ALT), GGT, and ALP were assayed by a biotechnical analyzer (BT 1500, Italy) at the Central Laboratory in the cohort center. Accuracy and precision of all methods were performed in accordance with the relevant guidelines and regulations.

### 2.3. Definition of Terms

Participants were grouped as having normal BP (untreated SBP <120 mmHg and DBP <80 mmHg), elevated BP (untreated SBP 120–129 mmHg and DBP <80 mmHg), stage 1 hypertension (untreated SBP 130–139 mmHg or DBP 80–89 mmHg), or stage 2 hypertension (SBP_140 mmHg or more, DBP_90 mmHg or more, or taking antihypertensive drugs) as defined by the 2017 American College of Cardiology (ACC)/American Heart Association (AHA) BP guideline [17]. The mean blood pressure was calculated by this formula: BP = (SBP + 2 DBP)/3.

Elevated serum ALT, AST, GGT, and ALP levels were defined according to the reference range of the laboratory in the cohort center. Elevated serum levels of ALT and AST were considered greater than 40 and 35 U/L in males and females, respectively. Elevated serum levels of GGT were defined as greater than 54 and 37 U/L in males and females, respectively. Elevated serum levels of ALP were defined as greater than 306 U/L in both genders. Also, subjects within the normal ranges of ALT, AST, GGT, and ALP were divided into the following quartiles: for ALT ≤14 U/L, 15–19 U/L, 20–25 U/L, and 26–40 U/L in males and ≤12 U/L, 13–15 U/L, 16–20 U/L, and 21–35 U/L in females; for AST ≤16 U/L, 17–19 U/L, 20–23 U/L, and 24–40 U/L in males and ≤14 U/L, 15-16 U/L, 17–20 U/L and 21–35 U/L in females. The quartiles for GGT were as follows: ≤18 U/L, 19–23 U/L, 24–31 U/L, and 32–54 U/L in males and ≤14 U/L, 15–18 U/L, 19–23 U/L, and 24–37 U/L in females. The quartiles for ALP were including ≤177 U/L, 178–209 U/L, 210–244 U/L, and 245–306 U/L in both genders.

### 2.4. Statistical Analyses

Quantitative variables were described as either the mean ± standard deviation or median [IQR] as appropriate and categorical variables as the frequency and percentage. Also, baseline characteristics of individuals were compared across the groups of high blood pressure (normal, elevated, stage 1 hypertension and stage 2 hypertension) and serum concentrations of liver enzymes, using the chi-square test for categorical variables and one-way ANOVA test for normally distributed quantitative variables and the Kruskal-Wallis test for nonnormally distributed quantitative variables.

Associations between blood pressure and liver enzymes were evaluated by crude and adjusted models in the sex-specific regression analysis, and confounder's variables were identified using relevant epidemiological texts and based on subject matter knowledge. Potential confounding variables were sequentially entered into models according to their hypothesized strengths of association with serum concentrations of liver enzymes and high blood pressure. To reach this goal, confounding variables with a *P* value <0.25 were selected as confounders. First, to investigate the associations using linear regression, the assumption of normality of the distribution of liver enzymes and linear correlation between those factors with blood pressure was tested using normal probability plots (skewness and kurtosis index) and scatter plots, respectively. Due to nonnormal distribution of data and to improve normality and linear correlation, log transformation was done for all liver serum enzymes in all analyses. The assumption of normality and linear correlation was valid for log-transformed variables except for GGT.

The associations between continuous values of blood pressure and log-transformed (except GGT classified by median) of each serum concentration of liver enzymes were evaluated by crude and multiple linear regression analysis. In linear regression models, the collinearity of the variables was examined by calculating the variance inflation factor using multiple linear regression analysis. Findings of the model indicated prominent collinearity between cholesterol and LDL cholesterol, and accordingly, we selected LDL cholesterol in the regression model for further analyses. Data were presented as unstandardized and standardized regression coefficients and 95% confidence intervals (CIs). In addition, we used dichotomous logistics regression analysis to estimate odds ratios (ORs) with 95% confidence intervals (CIs) for the association between hypertension and liver enzymes levels. The baseline model is stratified on the status of serum concentrations of liver enzymes. Adjusted model 1 was adjusted for age, gender, education years, and wealth status index. Adjusted model 2 has additional adjustments for cigarette smoking, alcohol drinking, opium consumption, BMI, and physical activity level. Adjusted model 3 has additional adjustments for diabetes, family history of hypertension in first-degree relatives, and family history of hypertension in second-degree relatives. Adjusted model 4 includes all variables considered in adjusted model 3, plus triglycerides, LDL, HDL, taking hepatotoxic drugs, and fatty liver.

In addition, logistic regression analysis was performed to assess the association between hypertension and the serum levels of liver enzymes within normal ranges. To reach this goal, levels of serum concentrations of liver enzymes were categorized into quartiles within normal ranges and elevated levels to test for possible dose-related response relationships. Finally, we used multinomial logistic regression models to strengthen the relationship between the level of liver enzymes with a stage of high blood pressure (elevated, stage 1 hypertension, and stage 2 hypertension). All analyses were performed through State V.12. All *P* values are two-sided, and *P* values <0.05 and 95% confidence intervals were considered statistically significant.

## 3. Results

In this study, 9930 participants in the baseline phase of the Rafsanjan adult cohort study who completed the medical questionnaire were included. From this population, 4623 (46.56%) were male and 5307 (53.44%) were female.  Table 1 shows some selected characteristics including sociodemographic, lifestyle, personal habits, anthropometric measures, clinical risk factors, and laboratory assessment in hypertensive and normotensive individuals. Based on the guidelines of the 2017 American College of Cardiology/American Heart Association (ACC/AHA) for blood pressure classification, the 9930 participants were categorized as having normal BP (*n* = 5656), elevated BP (*n* = 297), stage 1 hypertension (*n* = 1486), and stage 2 hypertension (*n* = 2491). Among all participants, the prevalence of elevated BP, stage 1 hypertension, and stage 2 hypertension was estimated at 2.99%, 14.96%, and 25.09, respectively. Elevated AST, ALT, GGT, and ALP were observed in %3, %8.94, % 10.24, and %9.79 of subjects, respectively.

Age, gender, education level, physical activity, alcohol and opium consumption, cigarette smoking, BMI, wealth score index, diabetes, family history of hypertension, fatty liver and taking hepatotoxic drugs, the levels of cholesterol, triglycerides, LDL, HDL, FBS, AST, ALT, GGT, and ALP had significant relationships with hypertension (Table 1, *P* < 0.001) while the mean level of HDL was not a significant variable for hypertension (Table 1, *P* > 0.05).

Table 2 shows the associations of the liver enzymes with the baseline variables, which are usually related to hypertension. A significant association was seen between all liver enzymes with physical activity, BMI, FBS, cholesterol, triglycerides, LDL cholesterol, and diabetes. The results showed that ALT and ALP were significantly related to alcohol and opium consumption. GGT and ALP had a significant association with cigarette smoking and hepatotoxic drugs. Serum ALT and GGT were associated with age, education, WSI, fatty liver, and the sex of the participants. Serum ALP was related to age, education, and WSI. Additionally, serum AST was related to fatty liver and alcohol consumption.

Table 3 shows the association between liver enzymes with systolic, diastolic, and mean blood pressures by linear regression analysis. Results without the confounder effect showed that ALT, AST, and GGT were positively associated with systolic, diastolic, and mean blood pressures in both genders, and ALP was positively associated with systolic and mean blood pressures in females. After adjusting the model, ALT and AST were positively associated with systolic, diastolic, and mean blood pressures in both genders, and ALP was positively associated with systolic and mean blood pressures in females. Significant associations were not observed regarding GGT after adjusting the model.

Table 4 presents the association of the serum levels of liver enzymes with abnormal blood pressure, using the crude and four adjusted models. As seen in Table 4, in the crude and adjusted model 1, elevated AST was associated with higher odds of abnormal blood pressure. The odds of abnormal blood pressure significantly increased along with the higher levels of ALT, GGT, and ALP in crude, adjusted models 1, 2, and 3, which remained significant only for ALP even in adjusted model 4 (OR:1.34, 95% CI = 1.16–1.56). After sex-specific analyses, an association of the elevated levels of liver enzymes with abnormal blood pressure remained significant only for ALP in the fully adjusted model in both males and females (OR in males: 1.36, 95% CI = 1.09–1.69, OR in females: 1.25, 95% CI = 1.01–1.54) (eTable 1). We evaluated the odds of abnormal blood pressure with respect to the serum levels of liver enzymes within normal ranges. In subjects with normal levels of ALT, AST, GGT, and ALP, in all models, dose-response increases were observed with the highest odds ratios in the 4th quartile for abnormal blood pressure. In the fully adjusted model, in subjects with elevated levels of ALT, AST, GGT, and ALP, the OR of abnormal blood pressure was 1.37 (1.14–1.64), 1.37 (1.04–1.80), 1.34 (1.12–1.60), and 1.53 (1.29–1.83), respectively, which was significantly higher than that of the subjects in normal quartile 1 (Table 4). In subjects with normal levels of ALT, AST, GGT, and ALP, dose-response increases were observed with the highest odds ratios in the 4th quartile for abnormal blood pressure in both males and females in the fully adjusted model (eTable 1).

Table 5 shows the association of the serum levels of liver enzymes with elevated blood pressure, stage 1 hypertension, and stage 2 hypertension in study participants using the crude and adjusted models. The crude model is stratified on the status of serum levels of liver enzymes. The adjusted model was adjusted for age, gender, education years, wealth status index, cigarette smoking, alcohol drinking, opium consumption, BMI, physical activity level, diabetes, family history of hypertension in first-degree relatives, family history of hypertension in second-degree relatives, triglycerides, LDL, HDL, taking hepatotoxic drugs, and fatty liver.

The odds of stage 1 hypertension increased along with the higher concentrations of ALT, GGT, and ALP, which remained significant for ALP even in the adjusted model (OR: 1.28, 95% CI = 1.05–1.57). As seen in Table 5, in the crude regression model, elevated ALP, elevated AST, and elevated GGT were associated with higher odds of stage 2 hypertension ((OR: 1.84, 95% CI = 1.58–2.14), (OR: 1.39, 95% CI = 1.07–1.82), and (OR: 1.60, 95% CI = 1.37–1.85), respectively), while, after adjusting for all mentioned variables, we found that, among liver enzymes, only elevated ALP was significantly correlated with the odds of stage 2 hypertension (OR: 1.37, 95% CI = 1.14–1.64).

Additionally, to evaluate the association of the serum levels of liver enzymes with elevated blood pressure, stage 1 hypertension, and stage 2 hypertension according to sex, sex-specific analyses were done (e Table 2). In the fully adjusted model, in subjects with normal levels of ALT, dose-response increases were observed only for stage 1 hypertension in both males and females. In subjects with normal levels of AST, dose-response increases were observed for stage 1 hypertension in females and for stage 2 hypertension in both males and females. In subjects with normal levels of GGT, dose-response increases were observed for elevated blood pressure in women and for stage 1 hypertension and stage 2 hypertension in both males and females. Furthermore, in subjects with normal levels of ALP, dose-response increases were observed for stage 1 hypertension in both men and females and for stage 2 hypertension in males (e Table 2).

## 4. Discussion

The prevalence of hypertension is increasing globally [13]. Early detection of hypertension as a major risk factor to cardiovascular diseases is of great importance, so finding a clear association between this disorder and liver enzymes may be helpful for the earlier prognosis of hypertension. The present study is a population-based study aimed to evaluate the association between serum levels of liver enzymes and hypertension in the participants of the Rafsanjan Cohort Study.

The main finding of this study was that there was a direct association between elevated serum concentrations of ALT, GGT, and ALP and an increased odd of hypertension even after adjusting for some potential confounding variables such as those related to demographic, lifestyle, history of diabetes, and family history of hypertension compared with the individual with normal levels of liver enzymes. When these analyses were adjusted for more confounding variables such as triglyceride, LDL cholesterol, HDL cholesterol levels, hepatotoxic drugs intake, and fatty liver, although no such significant association was observed for ALT and GGT, a positive association was significantly observed between serum ALP and hypertension. This result is consistent with some previous reports that suggest that increased levels of ALP may be associated with the risk of nonhepatic diseases, such as hypertension [10–12]. We also found that for analyses of associations between elevated serum concentrations of ALT, AST, GGT, and ALP and blood pressure, these confounding variables should be considered as potential confounders.

The association between serum levels of liver enzymes and hypertension has been less reported compared to other medical conditions such as metabolic syndrome and diabetes. A previous study conducted by the United States National Health and Nutrition Examination Survey on 4,155 men and women showed that ALP had a significant association with a higher frequency of hypertension [12]. Yuji Shimizu et al. showed that an elevated level of ALP was a significant risk factor of hypertension, for both male and female nondrinkers, but not for drinkers. They suggested that for analyses of associations between ALP and blood pressure, alcohol consumption should thus be considered as a potential confounder [11]. As was performed in the present study, nevertheless, this relationship was still observed. However, there is a possibility of the effect of residual confounding, because alcohol consumption is associated with social stigma in Iran (alcohol consumption is generally forbidden in Iran due to religious restriction), so there is a probability of underreporting of alcohol consumption in this population. For this reason and since alcohol consumption is rare among Iranian women, we examined these associations based on gender. Our study demonstrated that serum ALP level is positively associated with the prevalence of hypertension in both males and females.

Tehran Lipid and Glucose Study (TLGS) reported that increased levels of ALT, GGT, and ALP were positively associated with hypertension [10]. However, in this study, only confounders adjusted in models included gender, age, and BMI while, in our study, more confounders were examined to investigate the association between liver enzymes and hypertension. Also, a cross-sectional study in Bangladeshi adults showed that the prevalence of elevated liver enzymes was higher in hypertensive individuals. In the hypertensive group, GGT and ALP concentrations were higher in males and AST was higher in females but no difference was observed for ALT concentration between males and females. In a multivariate-adjusted logistic model, increased serum levels of ALT and GGT but not ALP were positively associated with hypertension in Bangladeshi adults [3]. Also, a population-based cross-sectional study in China reported that participants in the highest quintile of GGT levels had significantly higher odds for hypertension [8]. Our findings were consistent with the results of a previous relevant study among a similar population group [10]. However, the findings of the previous studies are contradictory among different population groups.

A contradiction between the results of our study and the aforementioned studies is possibly due to the fact that more confounders are considered in our study which could be a strength. Furthermore, in the above-mentioned studies, normal blood pressure was defined as an untreated blood pressure less than140/90 mmHg, but in our study, normal blood pressure was defined as an untreated blood pressure less than120/80 mmHg [17], so based on this modified definition, our findings indicated that, among liver enzymes, only elevated ALP was significantly correlated with the odds of stages 1 and 2 hypertension.

Although the exact mechanism underlying the association between increased serum levels of liver enzymes and risk of hypertension remains unclear [10, 11], some possible mechanisms are suggested by several studies including the following: a higher level of bone-type ALP activity may accelerate the development of hypertension through vascular calcification [18, 19], and impaired vascular homeostasis [20–22] or higher serum levels of ALP may be a risk factor for progression of atherosclerosis [23]. Most forms of total ALP are produced by bone and liver cells. The average ratio of bone-type ALP in total ALP is about 50%. Other isoenzymes of ALP include liver-type ALP (about 25%), biliary canaliculi-type ALP (about 10%), intestinal-type ALP (about 10%), and placental-type ALP (only 1%) [24].

In addition, the association between hypertension and serum concentrations of ALT, AST, GGT, and ALP within the normal range was assessed. Our findings showed that, in individuals with normal serum levels of ALT, AST, GGT, and ALP and those with a higher enzyme concentration, odds of high blood pressure was increasing with a dose-response relationship in both males and females. In study of Rahman, the elevated GGT, but not ALT, was associated with the presence of hypertension in males and females. For GGT, in the highest quartile group compared with the lowest quartile, the multivariate-adjusted OR for hypertension was 2.29 (1.68–3.14) in males and 1.52 (1.27–1.83) in females [3]. In agreement with the results of our study, some previous studies also showed that increased levels of ALP [10–12], GGT [8, 10], ALT [10, 25, 26], and AST [25, 27] within the normal range are associated with the odds of hypertension. For early detection of nonliver-related disorders such as hypertension, it seems logical that the cutoff level of serum ALT, AST, GGT, and ALP concentrations is modified at a lower level.

This study has strengths and limitations. One of the main strengths of our study is its population-based nature with a large sample size, extensive data collection for the exposure of interest (liver enzymes levels), and potential confounders (e.g., age, sex BMI, cigarette smoking, etc.). In the present study, new definitions for blood pressure classification by the 2017 American College of Cardiology (ACC)/American Heart Association (AHA) BP guideline were used which could be another strength. However, the cross-sectional design of the study did not allow deriving any causal inferences and the possible role of elevated liver enzyme levels in hypertension. Accordingly, it is suggested that this relationship be reconsidered in the follow-up phase of this prospective study. Furthermore, we did not have any ideas about the role of specific types of ALP in hypertension, because the ALP's isozymes were not detected in the study.

## 5. Conclusions

Increased serum ALP activity was positively associated with increased odds of hypertension. Based on the results of the present study, ALP could be helpful for the early detection of hypertension.

## Figures and Tables

**Table 1:** Demographic, selected medical and laboratory characteristics of study participants by blood pressure groups (*n* = 9930).

Characteristics	Overall (*n* = 9930)	Blood pressure groups				
		Normal (*n* = 5656)	Elevated (*n* = 297)	Stage 1 hypertension (*n* = 1486)	Stage 2 hypertension (*n* = 2491)	*P* value
Age-yr. no (%)				<0.001		
35–45	3694 (100)	2789 (75.50)	38 (1.03)	561 (15.19)	306 (8.28)	
46–55	3059 (100)	1699 (55.54)	75 (2.45)	499 (16.31)	786 (25.69)	
≥56	3176 (100)	1167 (36.74)	184 (5.79)	426 (13.41)	1399 (44.05)	
Mean ± SD	49.94 ± 9.56	47.13 ± 8.97	56.14 ± 8.39	49.41 ± 8.92	55.91 ± 8.22	<0.001
Gender-no (%)						<0.001
Female	5307 (100)	2964 (55.85)	116 (2.19)	634 (11.95)	1593 (30.02)	
Male	4623 (100)	2692 (58.23)	181 (3.92)	852 (18.43)	898 (19.42)	
Education-no (%)						<0.001
≤5 years	3484 (100)	1309 (47.04)	122 (3.50)	496 (14.24)	1227 (35.22)	
6–12 years	4817 (100)	2978 (61.82)	133 (2.76)	734 (15.24)	972 (20.18)	
≥13 years	1625 (100)	1035 (63.69)	42 (2.58)	256 (15.75)	292 (17.97)	
Physical activity-no (%)						<0.001
Low	2540 (100)	1309 (51.54)	87 (3.43)	393 (15.47)	751 (29.57)	
Moderate	4906 (100)	2832 (57.73)	121 (2.47)	713 (14.53)	1240 (25.28)	
Heavy	2484 (100)	1515 (60.99)	89 (3.58)	380 (15.30)	500 (20.13)	
Mean ± SD	38.80 ± 6.31	39.15 ± 6.55	38.90 ± 6.48	39.01 ± 6.73	37.85 ± 5.31	<0.001
BMI-no (%)						<0.001
<25	2866 (100)	2058 (71.81)	99 (3.45)	334 (11.65)	375 (13.08)	
25–29.9	4068 (100)	2319 (57.01)	122 (3.00)	637 (15.66)	990 (24.34)	
≥30	2990 (100)	1274 (42.61)	76 (2.54)	515 (17.22)	1125 (37.63)	
Mean ± SD	27.82 ± 4.92	26.77 ± 4.59	27.11 ± 4.46	28.62 ± 4.86	29.81 ± 5.01	<0.001
Wealth score index-no (%)						<0.001
Low	2335 (100)	1301 (55.72)	74 (3.17)	315 (13.49)	645 (27.62)	
Low-middle	2849 (100)	1546 (54.26)	101 (3.55)	447 (15.69)	755 (26.50)	
Middle-high	3974 (100)	2338 (58.83)	96 (2.42)	599 (15.07)	941 (23.68)	
High	764 (100)	463 (60.60)	26 (3.40)	125 (16.36)	150 (19.63)	
Alcohol consumption-no (%)						<0.001
Yes	993 (100)	654 (65.86)	27 (2.72)	170 (17.12)	142 (14.30)	
No	8918 (100)	4990 (55.95)	267 (2.99)	1316 (14.76)	2345 (26.30)	
Cigarette smoking-no (%)						<0.001
Yes	2544 (100)	1586 (62.34)	102 (4.01)	377 (14.82)	479 (18.83)	
No	7367 (100)	4058 (55.08)	192 (2.61)	1109 (15.05)	2008 (27.26)	
Opium consumption-no (%)						<0.001
Yes	2345 (100)	1446 (61.66)	98 (4.18)	335 (14.29)	466 (19.87)	
No	7566 (100)	4198 (55.49)	196 (2.59)	1151 (15.21)	2021 (26.71)	
Diabetes-no (%)						<0.001
Yes	1933 (100)	654 (33.83)	73 (3.78)	239 (12.36)	967 (50.03)	
No	7997 (100)	5002 (62.55)	224 (2. 80)	1247 (15.59)	1524 (19.06)	
Family history of hypertension in first-degree relatives-no (%)						<0.001
Yes	5469 (100)	2850 (52.11)	136 (2.49)	824 (15.07)	1524 (30.33)	
No	4461 (100)	2806 (62.90)	161 (3.61)	662 (14.84)	832 (18.65)	
Family history of hypertension in second-degree relatives-no (%)						0.019
Yes	1627 (100)	961 (59.07)	36 (2.21)	257 (15.80)	373 (22.93)	
No	8303 (100)	4695 (56.55)	261 (3.14)	1229 (14.80)	2118 (25.51)	

Characteristics	Overall (*n* = 9991)	Blood Pressure Groups				
		Normal (*n* = 5716)	Elevated (*n* = 297)	Stage 1 hypertension (*n* = 1486)	Stage 2 hypertension (*n* = 2491)	*P*-value
Fatty liver						<0.001
Yes	1013 (100)	483 (47.68)	20 (1.97)	138 (13.62)	372 (36.72)	
No	8917 (100)	5173 (58.01)	277 (3.11)	1348 (15.12)	2119 (23.76)	
Taking hepatotoxic drugs						<0.001
Yes	2387 (100)	858 (35.94)	81 (3.39)	267 (11.19)	1181 (49.48)	
No	7543 (100)	4798 (63.61)	216 (2.86)	1219 (16.16)	1310 (17.37)	
Cholesterol						
Mean ± SD	198.66 ± 38.05	196.19 ± 36.51	206.32 ± 40.03	205.30 ± 36.95	199.39 ± 41.13	<0.001
Triglycerides						
Median (interquartile range)	145 (106–199)	135 (99–189)	141.5 (110–196)	154 (115–214)	160 (119–217)	<0.001
LDL cholesterol						
Mean ± SD	108.17 ± 30.29	107.36 ± 28.81	116.05 ± 31.28	112.93 ± 30.15	106.23 ± 33	<0.001
HDL cholesterol						
Mean ± SD	57.75 ± 10.87	57.90 ± 10.89	56.97 ± 11.35	57.26 ± 10.49	57.80 ± 10.99	0.135
FBS						<0.001
Elevated	1669 (100)	594 (35.59)	80 (4.79)	209 (12.52)	786 (47.09)	
Mean ± SD	113.28 ± 39.09	107.18 ± 32.94	121.06 ± 43.18	111.16 ± 34.44	127.52 ± 49.05	<0.001
AST-no (%)						0.051
Elevated	294 (100)	145 (49.32)	8 (2.72)	51 (17.35)	90 (30.61)	
Median (interquartile range)	18 (15–22)	17 (15–21)	18 (15–22)	19 (15–23)	18 (15–22)	<0.001
ALT-no (%)						<0.001
Elevated	882 (100)	461 (52.27)	20 (2.27)	175 (19.84)	226 (25.42)	
Median (interquartile range)	18 (13–25)	17 (12–24)	18 (13–23)	20 (14–28)	19 (14–26)	<0.001
GGT-no (%)						<0.001
Elevated	1010 (100)	482 (47.72)	35 (3.47)	167 (16.53)	326 (32.28)	
Median (interquartile range)	21 (16–30)	20 (15–29)	22 (16.5–30)	23 (18–33)	23 (17–32)	<0.001
ALP-no (%)						<0.001
Elevated	966 (100)	440 (45.55)	32 (3.31)	156 (16.15)	338 (34.99)	
Median (interquartile range)	216 (181–259)	208 (175–250)	224.5 (190–266)	220 (186–265)	229 (190–275)	<0.001

Abbreviations: BMI: body mass index; FBS: fasting blood sugar; AST: aspartate aminotransferase; ALT: alanine aminotransferase; ALP: alkaline phosphatase; GGt: *γ*-glutamyl transferase.

**Table 2:** The associations of the liver enzymes with demographic and selected medical and laboratory characteristics in the study participants.

Characteristic	Elevated ALT	*P* value	Elevated AST	*P* value	Elevated GGT	*P* value	Elevated ALP	*P* value
Age cat-no. (%)		<0.001		0.905		<0.001		<0.001
35–45	438 (49.27)		112 (37.58)		310 (30.45)		215 (22.10)	
46–55	268 (30.15)		94 (31.54)		335 (32.91)		344 (35.35)	
≥56	183 (20.58)		92 (30.87)		373 (36.64)		414 (42.55)	
Gender-no. (%)		<0.001		0.621		0.001		0.827
Female	348 (39.15)		155 (52.01)		595 (58.45)		523 (9.85)	
Male	541 (60.85)		143 (47.99)		423 (41.55)		450 (46.25)	
Education-no. (%)		<0.001		0.090		<0.001		<0.001
≤5 years	255 (28.72)		122 (40.94)		422 (41.49)		463 (47.63)	
6–12 years	444 (50.00)		129 (43.29)		443 (43.56)		403 (41.46)	
≥13 years	189 (21.28)		47 (15.77)		152 (14.95)		106 (10.91)	
Physical activity-no. (%)		0.009		0.042		<0.001		0.006
Low	381 (42.86)		135 (45.30)		450 (44.20)		423 (43.47)	
Moderate	409 (46.01)		140 (46.98)		493 (48.43)		460 (47.28)	
Heavy	99 (11.14)		23 (7.72)		75 (7.37)		90 (9.25)	
BMI-no. (%)		<0.001		<0.001		<0.001		0.038
<25	120 (13.53)		53 (17.79)		167 (16.42)		249 (25.62)	
25–29.9	429 (48.37)		123 (41.28)		464 (45.62)		403 (41.46)	
≥30	338 (38.11)		122 (40.94)		386 (37.95)		320 (32.92)	
WSI-no. (%)		<0.001		0.137		<0.001		<0.001
Low	213 (23.99)		79 (26.51)		274 (26.94)		306 (31.48)	
Low-middle	247 (27.82)		90 (30.20)		316 (31.07)		276 (28.40)	
Middle-high	328 (36.94)		101 (33.89)		372 (36.58)		352 (36.21)	
High	100 (11.26)		28 (9.40)		55 (5.41)		38 (3.91)	
Alcohol consumption-no. (%)		<0.001		0.006		0.945		0.045
Yes	142 (16.15)		43 (14.78)		101 (10.05)		114 (11.83)	
No	737 (83.85)		248 (85.22)		904 (89.95)		850 (88.17)	
Cigarette smoking-no. (%)		0.311		0.441		0.049		<0.001
Yes	213 (24.23)		69 (23.71)		232 (23.08)		318 (32.99)	
No	666 (75.77)		222 (76.29)		773 (76.92)		646 (67.01)	
Opium consumption-no. (%)		0.001		0.227		0.583		<0.001
Yes	168 (19.11)		60 (20.62)		230 (22.89)		307 (31.85)	
No	711 (80.89)		231 (79.38)		775 (77.11)		657 (68.15)	
Diabetes-no. (%)		0.041		0.013		<0.001		<0.001
Yes	195 (10.10)		74 (3.83)		302 (15.66)		281 (14.56)	
No	1735 (89.90)		1856 (96.17)		1627 (84.34)		1649 (85.44)	
Family history of hypertension in first-degree relatives-no (%)		0.658		0.469		0.904		0.117
Yes	492 (55.78)		168 (3.08)		558 (10.24)		509 (52.69)	
No	390 (44.22)		126 (96.92)		4890 (89.76)		457 (47.31)	
Family history of hypertension in second-degree relatives-no (%)		0.643		0.024		0.038		0.003
Yes	149 (16.89)		34 (2.10)		142 (8.78)		125 (12.94)	
No	733 (83.11)		1583 (97.90)		1475 (91.22)		841 (87.06)	
Fatty liver-no. (%)		<0.001		<0.001		<0.001		0.409
Yes	167 (18.93)		53 (18.03)		165 (16.34)		106 (10.97)	
No	715 (81.07)		241 (81.97)		845 (83.66)		860 (89.03)	
Use of hepatotoxic drugs-no. (%)		0.207		0.254		<0.001		<0.001
Yes	197 (22.34)		79 (26.87)		315 (31.19)		304 (31.47)	
No	685 (77.66)		215 (73.13)		695 (68.81)		662 (68.53)	
Cholesterol-no. (%)		<0.001		0.003		<0.001		<0.001
Normal	394 (44.32)		141 (47.32)		433 (42.53)		487 (50.05)	
Elevated	495 (55.68)		157 (52.68)		585 (57.47)		486 (49.95)	
Triglycerides-no. (%)		<0.001		0.005		<0.001		<0.001
Normal	557 (62.65)		203 (68.12)		596 (58.55)		662 (68.04)	
Elevated	332 (37.35)		95 (31.88)		422 (41.45)		311 (31.96)	
LDL cholesterol-no. (%)		<0.001		<0.001		<0.001		0.001
Normal	633 (71.28)		201 (67.68)		699 (68.73)		712 (73.25)	
Elevated	255 (28.72)		96 (32.32)		318 (31.27)		260 (26.75)	
HDL cholesterol-no. (%)		0.855		0.479		0.679		0.594
Normal	885 (99.55)		296 (99.33)		1013 (99.51)		970 (99.69)	
Reduced	4 (0.45)		2 (0.67)		5 (0.49)		3 (0.31)	
FBS-no. (%)		<0.001		<0.001		<0.001		<0.001
Normal	677 (76.15)		217 (72.82)		696 (68.37)		682 (70.09)	
Elevated	212 (23.85)		81 (27.18)		322 (31.63)		291 (29.91)	

Abbreviations: BMI: body mass index; FBS: fasting blood sugar; AST: aspartate aminotransferase; ALT: alanine aminotransferase; ALP: alkaline phosphatase; GGt: *γ*-glutamyl transferase; WSI: Wealth score index.

**Table 3:** Sex-specific linear regression analysis of liver enzymes with systolic, diastolic, and mean blood pressures in the study participants (*n* = 9991).

Variables	SBP					DBP				Mean BP^*∗*^			
	Model	Unstandardized regression coefficient	Adjusted R2	Standardized regression coefficient	*P* value	Unstandardized regression coefficient	Adjust R2	Standardized regression coefficient	*P* value	Unstandardized regression coefficient	Adjust R2	Standardized regression coefficient	*P* value
Male													
Log-transformed ALT	Crude	2.65	0.08	0.089	<0.001	3.99	0.046	0.21	<0.001	3.54	0.028	0.17	<0.001
	Adjust^*∗∗*^	1.68	0.22	0.056	<0.001	1.52	0.17	0.081	<0.001	1.58	0.21	0.075	<0.001
Log-transformed AST	Crude	2.46	0.002	0.048	0.001	3.97	0.016	0.13	<0.001	3.46	0.009	0.097	<0.001
	Adjust	1.97	0.22	0.039	0.005	1.67	0.18	0.053	<0.001	1.77	0.21	0.05	<0.001
Log-transformed ALP	Crude	3.90	0.0036	0.062	<0.001	0.62	0.00	0.016	0.287	1.71	0.0013	0.038	0.009
	Adjust	2.78	0.22	0.044	0.001	0.73	0.18	0.019	0.172	1.43	0.21	0.032	0.016
GGT elevated	Crude	1.99	0.00	0.034	0.019	1.66	0.002	0.046	0.002	1.77	0.0017	0.044	0.003
	Adjust	0.56	0.22	0.01	0.469	0.095	0.18	0.003	0.846	0.28	0.21	0.0069	0.610
Female													
Log-transformed ALT	Crude	5.92	0.028	0.17	<0.001	3.14	0.023	0.15	<0.001	4.07	0.028	0.17	<0.001
	Adjust	1.73	0.29	0.049	<0.001	1.28	0.17	0.062	<0.001	1.44	0.24	0.06	<0.001
Log-transformed AST	Crude	6.37	0.015	0.12	<0.001	3.19	0.011	0.11	<0.001	4.25	0.014	0.12	<0.001
	Adjust	2.48	0.29	0.048	<0.001	1.75	0.17	0.058	<0.001	2.00	0.24	0.057	<0.001
Log-transformed ALP	Crude	13.73	0.053	0.23	<0.001	6.47	0.035	0.19	<0.001	8.89	0.048	0.22	<0.001
	Adjust	3.06	0.29	0.051	<0.001	2.52	0.17	0.073	<0.001	2.71	0.24	0.067	<0.001
GGT elevated	Crude	4.98	0.09	0.090	<0.001	2.32	0.005	0.072	<0.001	3.21	0.007	0.085	<0.001
	Adjust	0.32	0.028	0.006	0.626	0.46	0.16	0.014	0.264	0.43	0.23	0.011	0.354

Abbreviations: SBP: systolic blood pressure; DBP: diastolic blood pressure; BMI: body mass index; FBS: Fasting blood sugar; AST: aspartate aminotransferase; ALT: alanine aminotransferase; ALP: alkaline phosphatase; GGt: *γ*-glutamyl transferase. ^*∗*^ Mean BP = (SBP + 2 DBP)/3. ^*∗∗*^ Adjusted for confounding variables age (continuous variable), education years (continuous variable), wealth status index, the variables related to lifestyle (cigarette smoking, alcohol drinking and opium consumption), body mass index (continuous variable), physical activity level (continuous variable), diabetes (yes/no), family history of hypertension in first-degree relatives (yes/no), family history of hypertension in second-degree relatives (yes/no), triglycerides (continuous variable), LDL cholesterol (continuous variable), HDL cholesterol (continuous variable), hepatotoxic drugs (yes/no), and fatty liver (yes/no).

**Table 4:** Association of the serum levels of liver enzymes with abnormal blood pressure in study participants (*n* = 9991).

Liver enzyme	Crude model	Adjusted model 1	Adjusted model 2	Adjusted model 3	Adjusted model 4
	OR (95% Ci)^a^	OR (95% Ci)^b^	OR (95% Ci)^c^	OR (95% Ci)^d^	OR (95% Ci)^e^
ALT					
Normal	1	1	1	1	1
Elevated	1.22 (1.06–1.40)	1.65 (1.42–1.91)	1.27 (1.09–1.48)	1.23 (1.05–1.43)	1.16 (0.99–1.36)
Quartile 1	1	1	1	1	1
Quartile 2	1.38 (1.23–1.56)	1.38 (1.22–1.57)	1.11 (0.98–1.27)	1.10 (0.96–1.26)	1.07 (0.93–1.23)
Quartile 3	1.88 (1.67–2.11)	1.86 (1.64–2.11)	1.38 (1.21–1.57)	1.34 (1.18–1.53)	1.27 (1.11–1.45)
Quartile 4	2.00 (1.78–2.25)	2.22 (1.96–2.51)	1.53 (1.34–1.75)	1.47 (1.29–1.68)	1.36 (1.19–1.56)
Elevated	1.81 (1.55–2.00)	2.52 (2.13–2.97)	1.60 (1.34–1.90)	1.52 (1.27–1.81)	1.37 (1.14–1.64)
AST					
Normal	1	1	1	1	1
Elevated	1.34 (1.07–1.69)	1.42 (1.11–1.82)	1.22 (0.95–1.58)	1.20 (0.93–1.56)	1.15 (0.89–1.50)
Quartile 1	1	1	1	1	1
Quartile 2	1.16 (1.03–1.30)	1.14 (1.01–1.29)	1.11 (0.98–1.26)	1.19 (1.05–1.36)	1.17 (1.03–1.33)
Quartile 3	1.37 (1.23–1.53)	1.31 (1.16–1.47)	1.22 (1.08–1.38)	1.30 (1.15–1.47)	1.25 (1.11–1.42)
Quartile 4	1.52 (1.36–1.71)	1.56 (1.38–1.76)	1.35 (1.19–1.53)	1.44 (1.26–1.63)	1.36 (1.19–1.55)
Elevated	1.65 (1.30–2.10)	1.73 (1.34–2.24)	1.42 (1.09–1.85)	1.46 (1.11–1.91)	1.37 (1.04–1.80)
GGT					
Normal	1	1	1	1	1
Elevated	1.49 (1.31–1.70)	1.40 (1.22–1.61)	1.23 (1.07–1.42)	1.19 (1.03–1.38)	1.12 (0.96–1.30)
Quartile 1	1	1	1	1	1
Quartile 2	1.73 (1.55–1.95)	1.65 (1.45–1.87)	1.36 (1.19–1.56)	1.31 (1.14–1.50)	1.25 (1.01–1.43)
Quartile 3	2.00 (1.77–2.26)	1.91 (1.68–2.17)	1.46 (1.27–1.67)	1.40 (1.22–1.61)	1.31 (1.14–1.50)
Quartile 4	2.20 (1.95–2.40)	2.17 (1.91–2.46)	1.54 (1.34–1.76)	1.14 (1.23–1.62)	1.27 (1.10–1.46)
Elevated	2.46 (2.12–2.85)	2.24 (1.91–2.63)	1.64 (1.39–1.94)	1.52 (1.29–1.81)	1.34 (1.12–1.60)
ALP					
Normal	1	1	1	1	1
Elevated	1.64 (1.44–1.88)	1.36 (1.19–1.57)	1.41 (1.22–1.64)	1.40 (1.20–1.62)	1.34 (1.16–1.56)
Quartile 1	1	1	1	1	1
Quartile 2	1.31 (1.16–1.48)	1.19 (1.05–1.35)	1.12 (0.98–1.28)	1.13 (0.99–1.29)	1.11 (0.97–1.27)
Quartile 3	1.60 (1.42–1.81)	1.33 (1.17–1.51)	1.18 (1.03–1.35)	1.18 (1.03–1.35)	1.14 (1.00–1.31)
Quartile 4	1.92 (1.70–2.17)	1.51 (1.33–1.72)	1.39 (1.20–1.58)	1.36 (1.18–1.56)	1.29 (1.12–1.48)
Elevated	2.33 (2.00–2.72)	1.71 (1.46–2.02)	1.66 (1.40–1.97)	1.63 (1.37–1.94)	1.53 (1.29–1.83)

^a^The baseline model is stratified on the status of serum levels of liver enzymes. ^b^Adjusted model 1 is adjusted for confounding variables age (continuous variable), gender (male/female), education years (continuous variable), and wealth status index. ^C^Adjusted model 2 has additional adjustment for confounding the variables related to lifestyle (cigarette smoking, alcohol drinking and opium consumption), body mass index (continuous variable), and physical activity level (continuous variable). ^d^Adjusted model 3 has additional adjustment for diabetes (yes/no), family history of hypertension in first-degree relatives (yes/no), and family history of hypertension in second-degree relatives (yes/no). ^e^Adjusted model 4 has additional adjustment for triglycerides (continuous variable), LDL cholesterol (continuous variable), HDL cholesterol (continuous variable), hepatotoxic drugs (yes/no), and fatty liver (yes/no). Abnormal blood pressure was defined as SBP ≥120 mmHg or DBP ≥80 mmHg or taking antihypertensive drugs.

**Table 5:** Association of the serum levels of liver enzymes with elevated blood pressure, stage 1 hypertension, and stage 2 hypertension in study participants (*n* = 9991).

Liver enzyme	Elevated blood pressure		Stage 1 hypertension		Stage 2 hypertension	
	Crude model	Adjusted model	Crude model	Adjusted model	Crude model	Adjusted model
ALT						
Normal	1	1	1	1	1	1
Elevated	0.81 (0.51–1.28)	0.92 (0.57–1.51)	1.49 (1.24–1.79)	1.13 (0.93–1.37)	1.11 (0.94–1.31)	1.19 (0.98–1.45)
AST						
Normal	1	1	1	1	1	1
Elevated	1.03 (0.50–2.12)	0.96 (0.44–2.10)	1.32 (0.96–1.83)	1.11 (0.79–1.56)	1.39 (1.07–1.82)	1.21 (0.88–1.66)
GGT						
Normal	1	1	1	1	1	1
Elevated	1.42 (0.99–2.04)	1.21 (0.82–1.77)	1.34 (1.12–1.62)	1.11 (0.91–1.34)	1.60 (1.37–1.85)	1.09 (0.91–1.30)
ALP						
Normal	1	1	1	1	1	1
Elevated	1.42 (0.97–2.07)	1.10 (0.74–1.63)	1.38 (1.14–1.67)	1.28 (1.05–1.57)	1.84 (1.58–2.14)	1.37 (1.14–1.64)

Crude model: the baseline model is stratified on the status of serum levels of liver enzymes. Adjusted model: the adjusted model is for confounding variables age (continuous variable), gender (male/female), education years (continuous variable), wealth status index, lifestyle (cigarette smoking, alcohol drinking and opium consumption), body mass index (continuous variable), physical activity level (continuous variable), diabetes (yes/no), family history of hypertension in first-degree relatives (yes/no), family history of hypertension in second-degree relatives (yes/no), triglycerides (continuous variable), LDL cholesterol (continuous variable), HDL cholesterol (continuous variable), taking hepatotoxic drugs (yes/no), and fatty liver (yes/no). Subjects were grouped as having normal BP (untreated SBP <120 mmHg and DBP <80 mmHg), elevated BP (untreated SBP 120–129 mmHg and DBP <80 mmHg), stage 1 hypertension (untreated SBP 130–139 mmHg or DBP 80–89 mmHg), or stage 2 hypertension (SBP ≥140 mmHg, DBP ≥90 mmHg, or taking antihypertensive drugs).

## Data Availability

The datasets used during the current study are available on the PERSIAN Adult Cohort Study Center, Rafsanjan University of Medical Sciences, Iran. The data are not available publicly. However, upon reasonable request, the data can be obtained from the authors.

## Supplementary Materials

Supplementary Materials Additional file 1: eTable 1. Additional file 2: eTable 2.
