# Supplementary material for: Evaluation of Relationship between Serum Liver Enzymes and Hypertension: A Cross-Sectional Study Based on Data from Rafsanjan Cohort Study
Source: Int J Hypertens. 2022 Apr 14;2022:5062622. doi: 10.1155/2022/5062622 (PMC9023230; doi:10.1155/2022/5062622)
Supplement: Supplementary Materials — Additional file 1: eTable 1. Additional file 2: eTable 2. [file 5062622.f1.zip › 5062622.f1/eTable 1.sup.doc]

| eTable 1. Association of the levels of liver enzymes with abnormal blood pressure in study participants according to sex (n= 9991). | | |
| --- | --- | --- |
| **liver enzyme** | **Adjusted model in male** | **Adjusted model in female** |
|  | **OR (95%Ci)** | **OR(95%Ci)** |
| **ALT** | |  |
| Normal | 1 | 1 |
| Elevated | 1.18(0.96-1.45) | 0.96(0.75-1.24) |
| Quartile 1 | 1 | 1 |
| Quartile 2 | 1.13(0.93-1.38) | 0.97(0.80-1.18) |
| Quartile 3 | 1.31(1.06-1.61) | 1.13(0.94-1.36) |
| Quartile 4 | 1.28(1.03-1.58) | 1.29(1.07-1.55) |
| Elevated | 1.42(1.10-1.84) | 1.06(0.81-1.40) |
| **AST** | |  |
| Normal | 1 | 1 |
| Elevated | 1.16(0.79-1.70) | 1.07(0.74-1.54) |
| Quartile 1 | 1 | 1 |
| Quartile 2 | 1.17(0.97-1.41) | 1.12(0.92-1.35) |
| Quartile 3 | 1.24(1.030-1.49) | 1.16(0.98-1.38) |
| Quartile 4 | 1.26(1.04-1.53) | 1.31(1.09-1.50) |
| Elevated | 1.36(0.91-2.00) | 1.22(0.83-1.78) |
| **GGT** | |  |
| Normal | 1 | 1 |
| Elevated | 1.12(0.89-1.40) | 1.02(0.83-1.25) |
| Quartile 1 | 1 | 1 |
| Quartile 2 | 1.20(0.98-1.46) | 1.25(1.03-1.51) |
| Quartile 3 | 1.18(0.97-1.45) | 1.35(1.11-1.65) |
| Quartile 4 | 1.27(1.03-1.56) | 1.14(0.93-1.39) |
| Elevated | 1.32(1.01-1.73) | 1.21(0.95-1.54) |
| **ALP** | |  |
| Normal | 1 | 1 |
| Elevated | 1.36(1.09-1.69) | 1.25(1.01-1.54) |
| Quartile 1 | 1 | 1 |
| Quartile 2 | 1.11(0.90-1.35) | 1.09(0.90-1.31) |
| Quartile 3 | 1.14(0.93-1.39) | 1.11(0.91-1.34) |
| Quartile 4 | 1.27(1.04-1.56) | 1.24(1.02-1.51) |
| Elevated | 1.55(1.19-2.00) | 1.39(1.08-1.77) |
| The adjusted model is adjusted for confounding variables age (continuous variable), education years (continuous variable), wealth status index, cigarette smoking, alcohol drinking, opium consumption), body mass index (continuous variable), physical activity level (continuous variable), diabetes (yes/no), family history of hypertension in first-degree relatives (yes/no), family history of hypertension in second-degree relatives (yes/no), Triglycerides (continuous variable), LDL cholesterol (continuous variable), HDL cholesterol (continuous variable), hepatotoxic drugs (yes/no) and fatty liver (yes/no).  Abnormal blood pressure was defined as SBP ≥ 120 mmHg or DBP ≥ 80 mmHg or taking antihypertensive drugs. | | |
