# Supplementary material for: Evaluation of Relationship between Serum Liver Enzymes and Hypertension: A Cross-Sectional Study Based on Data from Rafsanjan Cohort Study
Source: Int J Hypertens. 2022 Apr 14;2022:5062622. doi: 10.1155/2022/5062622 (PMC9023230; doi:10.1155/2022/5062622)
Supplement: Supplementary Materials — Additional file 1: eTable 1. Additional file 2: eTable 2. [file 5062622.f1.zip › 5062622.f1/eTable 2.sup.doc]

| eTable 2. Association of the levels of liver enzymes with elevated blood pressure, stage 1 hypertension and stage 2 hypertension in study participants according to sex (n= 9991). | | | | | | |
| --- | --- | --- | --- | --- | --- | --- |
| **liver enzyme** | **Elevated blood pressure** | | **Stage 1 hypertension** | | **Stage 2 hypertension** | |
|  | Adjusted model | | Adjusted model | | Adjusted model | |
|  | men | women | men | women | men | Women |
| **ALT** |  |  |  |  |  |  |
| Normal | 1 | 1 | 1 | 1 | 1 | 1 |
| Elevated | 1.24(0.69-2.21) | 0.48(0.17-1.34) | 1.14(0.90-1.46) | 1.05(0.75-1.48) | 1.19(0.90-1.58) | 0.98(0.73-1.30) |
| Quartile 1 | 1 | 1 | 1 | 1 | 1 | 1 |
| Quartile 2 | 1.20(0.79-1.82) | 0.99(0.54-1.80) | 1.26(0.98-1.64) | 1.10(0.85-1.43) | 0.99(0.75-1.29) | 0.90(0.71-1.14) |
| Quartile 3 | 0.98(0.61-1.60) | 1.08(0.61-1.90) | 1.48(1.14-1.93) | 1.15(0.89-1.49) | 1.21(0.92-1.58) | 1.13(0.91-1.40) |
| Quartile 4 | 0.81(0.47-1.39) | 1.63(0.95-2.80) | 1.38(1.06-1.81) | 1.40(1.09-1.81) | 1.30(0.98-1.71) | 1.22(0.98-1.51) |
| Elevated | 1.22(0.63-2.37) | 0.57(0.19-1.68) | 1.52(1.11-2.08) | 1.22(0.84-1.78) | 1.38(0.98-1.94) | 1.04(0.76-1.43) |
| **AST** |  |  |  |  |  |  |
| Normal | 1 | 1 | 1 | 1 | 1 | 1 |
| Elevated | 0.88(0.27-2.88) | 1.09(0.38-3.11) | 1.17(0.75-1.83) | 1.00(0.59-1.69) | 1.16(0.70-1.92) | 1.13(0.75-1.71) |
| Quartile 1 | 1 | 1 | 1 | 1 | 1 | 1 |
| Quartile 2 | 1.30(0.85-1.97) | 0.61(0.31-1.21) | 1.08(0.85-1.36) | 1.14(0.88-1.49) | 1.24(0.98-1.59) | 1.14(0.91-1.43) |
| Quartile 3 | 1.34(0.87-2.06) | 1.36(0.82-2.24) | 1.20(0.95-1.51) | 1.24(0.98-1.57) | 1.26(0.98-1.60) | 1.10(0.89-1.35) |
| Quartile 4 | 1.06(0.64-1.74) | 1.02(0.58-1.80) | 1.24(0.98-1.58) | 1.35(1.05-1.74) | 1.32(1.02-1.70) | 1.33(1.08-1.65) |
| Elevated | 1.03(0.30-3.47) | 1.13(0.38-3.40) | 1.33(0.83-2.13) | 1.17(0.68-2.02) | 1.40(0.83-2.36) | 1.27(0.83-1.96) |
| **GGT** |  |  |  |  |  |  |
| Normal | 1 | 1 | 1 | 1 | 1 | 1 |
| Elevated | 1.54(0.91-2.59) | 1.00(0.56-1.79) | 1.10(0.84-1.45) | 1.08(0.81-1.43) | 1.06(0.78-1.43) | 0.99(0.79-1.25) |
| Quartile 1 | 1 | 1 | 1 | 1 | 1 | 1 |
| Quartile 2 | 1.10(0.70-1.73) | 1.93(1.10-3.41) | 1.37(1.07-1.76) | 1.31(1.01-1.70) | 1.06(0.81-1.39) | 1.18(0.92-1.48) |
| Quartile 3 | 1.08(0.68-1.73) | 1.12(0.58-2.14) | 1.25(0.97-1.61) | 1.38(1.04-1.81) | 1.13(0.86-1.48) | 1.37(1.08-1.74) |
| Quartile 4 | 1.11(0.67-1.83) | 0.98(0.51-1.89) | 1.24(0.95-1.62) | 1.17(0.88-1.56) | 1.36(1.04-1.79) | 1.13(0.89-1.43) |
| Elevated | 1.66(0.91-3.05) | 1.25(0.60-2.58) | 1.36(0.97-1.89) | 1.31(0.93-1.84) | 1.23(0.86-1.76) | 1.16(0.88-1.55) |
| **ALP** |  |  |  |  |  |  |
| Normal | 1 | 1 | 1 | 1 | 1 | 1 |
| Elevated | 1.36(0.83-2.23) | 0.77(0.40-1.49) | 1.27(0.97-1.67) | 1.31(0.97-1.76) | 1.46(1.10-1.94) | 1.27(1.01-1.61) |
| Quartile 1 | 1 | 1 | 1 | 1 | 1 | 1 |
| Quartile 2 | 1.15(0.70-1.87) | 0.65(0.35-1.20) | 1.06(0.72-1.37) | 1.28(0.98-1.66) | 1.15(0.88-1.50) | 1.02(0.81-1.28) |
| Quartile 3 | 1.01(0.62-1.66) | 0.94(0.54-1.64) | 1.23(0.96-1.57) | 1.40(1.07-1.84) | 1.09(0.83-1.42) | 0.96(0.76-1.22) |
| Quartile 4 | 1.19(0.72-1.95) | 0.86(0.49-1.51) | 1.30(1.01-1.67) | 1.58(1.20-2.07) | 1.29(0.98-1.69) | 1.09(0.86-1.37) |
| Elevated | 1.48(0.81-2.70) | 0.66(0.31-1.40) | 1.47(1.06-2.03) | 1.73(1.22-2.44) | 1.66(1.19-2.33) | 1.29(0.97-1.71) |
| The adjusted model is for confounding variables age (continuous variable), education years (continuous variable), wealth status index, lifestyle (cigarette smoking, alcohol drinking and opium consumption), body mass index (continuous variable), physical activity level (continuous variable), diabetes (yes/no), family history of hypertension in first-degree relatives (yes/no), family history of hypertension in second-degree relatives (yes/no), triglycerides (continuous variable), LDL cholesterol (continuous variable), HDL cholesterol (continuous variable), taking hepatotoxic drugs (yes/no) and fatty liver (yes/no).  Subjects were grouped as having normal BP (untreated SBP <120mmHg and DBP <80mmHg); elevated BP (untreated SBP 120-129mmHg and DBP <80mmHg); stage 1 hypertension (untreated SBP 130-139mmHg or DBP 80-89mmHg); or stage 2 hypertension (SBP ≥140mmHg, DBP≥90mmHg, or taking antihypertensive drugs). | | | | | | |
